# Patient Radiation Doses in Interventional Cardiology Procedures

**DOI:** 10.2174/157340309787048059

**Published:** 2009-01

**Authors:** Ioannis Pantos, Georgios Patatoukas, Demosthenes G Katritsis, Efstathios Efstathopoulos

**Affiliations:** 1 Department of Cardiology, Athens Euroclinic, Athens, Greece; 2 2^nd^ Department of Radiology, Medical Physics Unit, University of Athens, Athens, Greece

**Keywords:** Patient dosimetry, interventional cardiology.

## Abstract

Interventional cardiology procedures result in substantial patient radiation doses due to prolonged fluoroscopy time and radiographic exposure. The procedures that are most frequently performed are coronary angiography, percutaneous coronary interventions, diagnostic electrophysiology studies and radiofrequency catheter ablation. Patient radiation dose in these procedures can be assessed either by measurements on a series of patients in real clinical practice or measurements using patient-equivalent phantoms. In this article we review the derived doses at non-pediatric patients from 72 relevant studies published during the last 22 years in international scientific literature. Published results indicate that patient radiation doses vary widely among the different interventional cardiology procedures but also among equivalent studies. Discrepancies of the derived results are patient-, procedure-, physician-, and fluoroscopic equipmentrelated. Nevertheless, interventional cardiology procedures can subject patients to considerable radiation doses. Efforts to minimize patient exposure should always be undertaken.

## INTRODUCTION

The medical use of ionizing radiation, while offering great benefit to patients, also contributes significantly to radiation exposure of individuals and populations [1-3]. Interventional radiology and interventional cardiology (IC) contributes a significant proportion of the collective dose of the population from medical exposures. According to the results published by the United Nations Scientific Committee on the Effects of Atomic Radiation, interventional procedures contribute only 1% to the frequency of radiation use on the medical field whereas their contribution to collective dose is 10% [4]. When complex procedures are performed or procedures are repeated for the same patient, high radiation dose levels can occur because procedures often require long fluoroscopy time and a large number of images.

Over the last 10 years the number of IC procedures has increased rapidly [5, 6]. The main reasons is that IC permits specialists to avoid complicated invasive surgery which some patients might not tolerate due to factors of age or pathology [7] and results in limited hospitalization [5]. Additionally, knowledge of the benefits of IC becomes more widely spread and more complicated procedures are technically possible. Nevertheless, population dose and associated health risks are also increasing. The potential of occurrence of deterministic effects (injury of radiation, the severity of which varies with the dose and for which a dose threshold exists), especially to the skin, has been a subject of great concern. Additionally, estimation of the health risk owing to stochastic effects of radiation (malignant disease and heritable effects without a threshold level of dose, whose probability is proportional to the dose and whose severity is independent of the dose) especially for the younger patients is also under thorough investigation.

Reviewing the various studies on patient dosimetry in IC, it is evident that there is great variability in both methods of measurement and levels of radiation dose received by the patient. Some of the factors responsible for dose variability are the complexity of the procedure, operator experience, level of training in radiation protection, and type and performance of x-ray equipment available in the catheterization laboratory.

The purpose of this study is to review published literature on patient dosimetry in IC, discuss discrepancies of results, and comment on risks to patients and strategies to minimize patient radiation doses.

### Patient Dosimetry Review in IC

Data were collected from 72 studies published in international scientific literature between 1986 and 2008 concerning non-pediatric patient dose measurements and calculations in IC. Cardiac procedures reported in these studies fall into nine categories which are shown in Table **1** together with the corresponding number of studies.

The most frequently reported procedures were coronary angiography (CA) and percutaneous coronary intervention (PCI), followed by radiofrequency ablation (RFA) and combined CA and PCI. Other procedures for which patient dosimetric data were reported were CA with left ventriculography, electrophysiology studies, PCI with stent implantation, CA with PCI and stent implantation, and CA with left ventriculography and stent implantation. Many studies reported data for more than one procedure whereas some studies compared the dosimetric data for the same procedure under varying conditions (e.g. procedural technique, equipment, cardiologist’s experience etc).

The quantities used to assess patient dosimetry in these studies are tabulated in Table **2**.

The most commonly used quantities were fluoroscopy time and DAP followed by the number of cine frames and effective dose. Other quantities assessed were cine time, skin dose and coronary dose, the dose to a coronary artery measured by a catheter based dosimeter during irradiation [28]. Fluoroscopy time (usually measured in minutes) is a non-dosimetric quantity; however it is widely used to evaluate patient dosimetry since it is readily available and still the only dose metric routinely employed in many interventional laboratories. Nevertheless it does not incorporate information about dose rate and skin entrance ports [77, 78]. DAP (measured in Gy·cm^2^) is the product of the dose in air in a given plane by the area of the irradiating beam and is independent of the distance from the x-ray source because the decrease in dose with distance parallels the increase in area [79]. DAP is the initial quantity not only for estimating patient skin dose but for first establishing the stochastic risk to patients, characterized by effective dose (E) [42]. DAP is measured by an ionization chamber incorporated into the x-ray equipment and includes field non-uniformity effects such as anode-heel effect and use of beam-equalizing shutters (lung shutter). However it does not provide any information regarding the spatial distribution of the entrance beam on patient’s skin [77]. Cine time and cine frames (measured in seconds and number respectively) are also non-dosimetric quantities which are readily available but have the same limitations as fluoroscopy time. Effective dose (measured in mSv) introduced by the International Commission on Radiological Protection [80] is widely used as a stochastic risk related factor to assess radiation detriment and is also used to set dose limits and constraints, to limit the risk of cancer and hereditary effects. Effective dose can be assessed by three methods: (1) measurements in physical anthropomorphic phantoms using thermoluminescent dosimeters (TLDs), (2) by multiplying DAP by a conversion factor. Multiple sources of conversion factors exist but the most widely used are those proposed by the National Radiological Protection Board (NRPB) [81] and (3) by Monte Carlo based computer simulation codes such as WinODS [82], PCXMC [83] and XDOSE [84] which are fed with data of DAP for each projection, tube potential, field sizes and patient data. Skin dose is usually assessed by peak skin dose (measured in Gy) which is the highest absorbed dose received by any location on the patient’s skin and is thought to be best predictor of skin injury. Skin dose can be assessed with different methodologies [77]: (1) by direct calculation with off-line or on-line techniques, (2) by direct measurements on the patient with point detectors, (3) by direct measurement on the patient with large area detectors (e.g. Films). Coronary dose (measured in Gy) is measured by a TLD dosimeter placed at the tip of a catheter which is then advanced at the vicinity of the coronary artery subjected to angiography or intervention [28].

Patient dosimetry methodologies and quantities can be divided into three categories according to dosimetry objective (Table **2**) [85]: (i) dosimetry for stochastic risk evaluation, (ii) dosimetry for quality assurance and (iii) dosimetry to prevent deterministic effects of radiation. Effective dose (E) is the most suitable indicator in the assessment of diagnostic practice and population exposure and estimates the health risk owing to stochastic effects of radiation. DAP, fluoroscopy time, cine time and number of cine images are useful indicators to evaluate optimization level of radiological practise, to compare performance of equipment and operator skill or to compare the practice among different centres. Peak skin dose is important to assess the potential of deterministic effects due to skin irradiation and to prevent them. The literature of case reports, describing radiation induced skin injuries due to IC procedures is growing [86]. Therefore the potential of deterministic effects in some procedures may be of more concern than stochastic risk [85]. Coronary dose received during IC procedures although not frequently reported has been proposed as an iatrogenic component to coronary artery restenosis [87] and therefore might be of importance.

### Published Results

Published results on dosimetry in IC are presented in Table **3**. In order to calculate the mean of the reported average and median values for each IC procedures, the average and median value of each study was considered against the total number of patients included in the particular study. The total sample for each IC procedure and the sample used to calculate the mean values are also shown in Table **3**. The range of values shown in the table is the entire range considering the range of values reported in each study. The mean fluoroscopy time, mean DAP and mean effective dose are graphically presented in Fig. (**1**). Mean cine frames and mean cine time per procedure are presented in Fig. (**2**) while mean peak skin dose and mean coronary dose are presented in Fig. (**3**).

## DISCUSSION

It is evident from the tabulated data that the range of the reported values for each IC procedure is considerably wide. For example the range of DAP values for CA (1.1-2400 Gy·cm^2^) comprises the DAP values reported for almost any IC procedure. This is however due to the fact the CA is the most extensively studied procedure and dosimetric data include results reported in the 80s and 90s when radiation protection and catheterization laboratory equipment were less advanced. A recent study collected DAP values for 2265 coronary angiographies performed between 2003-2005 in seven centers and has also reported large variability of results although DAP values spanned in a narrower range of 5-130 Gy·cm^2^ [88]. The wide range of reported values are evident in all IC procedures and can be attributed to operator experience [40], workload [61], use of radiation-reducing techniques [38], procedural complexity [60, 89], examination technique [11] and catheterization laboratory equipment [42, 90]. In order to compare results between older and newer studies, mean DAP, mean fluoroscopy time and mean effective dose were calculated for results published before and after the year 2000 concerning the most extensively studied procedures (CA and PCI) (Table **4**). It is evident that due to on-going development in radiation protection and catheterization laboratory equipment there is considerable reduction in radiation received by patients.

The calculated mean values of the dosimetric and non-dosimetric quantities indicate that PCI procedures either with or without stent implantation result in increased radiation received by patients compared to angiography. When coronary angiography is combined with left ventriculography DAP and E are similar, or even slightly reduced in the combined procedures. However this is probably due to the fact that considerably lesser studies included ventriculography (8 studies, 1297 total patients) than CA without ventriculography (51 studies, 9100 total patients) and thus the calculated mean values are less representative, particularly for E which is calculated only in one study. The same applies for the particularly high fluoroscopy time and effective dose reported for procedures that combine CA with left ventriculography and stenting since these values result from a single study. Contradictory results on mean effective dose at PCI (sample 500, mean E=17 mSv) and CA with PCI (sample 147, E=13,6 mSv) are observed since it is expected that the combined procedure would result in higher effective dose. A likely explanation is that two studies on PCI with a large patient cohort (total 220 patients) are reporting effective dose values of 23.2 mSv [34] and 15,3 mSv [50] which are particularly high compared to other studies [34] and thus their contribution to mean effective dose results in an overall high mean value. Other studies on PCI with smaller patient cohort (total 22 patients) are reporting substantially lower effective dose values of 6.6 mSv [28] and 6.9 mSv [22]. EF studies generally result in low patient exposure since fluoroscopy is exclusively used (without image acquisition) while RFA ablation is the procedure where the maximum values of DAP and effective dose are reported owing to the extended fluoroscopic times (average fluoroscopic time 45.8 min).

A few studies have included measurements of coronary dose, the dose received by the coronary vessels during irradiation. The method of measurement involves the insertion of a catheter with a TLD dosimeter at its tip which is then advanced to the sinus of Valsalva corresponding to the coronary artery subjected to angiography or intervention [28]. The obvious advantage of the method is that it allows direct measurement of coronary dose, although this is not absolutely correct since due to technical and ethical reasons the dosimeter is not advanced inside the artery. Such a measurement might be important since experimental studies have shown that external beam radiation after stent implantation increases the restenosis rate [91, 92]. However the measured coronary doses were found approximately 2 orders of magnitude lower than the doses that have resulted in neointimal hyperplasia after external beam irradiation [93]. The main disadvantage is the invasive nature of the technique which makes it difficult to adopt in everyday clinical practice.

A number of studies have evaluated the effect of various parameters on patient dose at IC procedures. The parameters that have been investigated include catheterization laboratory equipment, procedural complexity, operator experience, and irradiation parameters. Broadhead *et al.* [20] compared patient dosimetry in two cardiology rooms, one equipped with biplane image intensifier system and one with single intensifier system, and found that the biplane system provides greater imaging capability but also increases patient dose. Bernardi *et al.* [57] and Padovani *et al.* [60] investigated the effect of the complexity of PCI procedures on patient exposure by dividing the procedures in ‘simple’, ‘medium’ and ‘complex’ based on a set of indexes and they found correlation between patient dose and procedural complexity. Arthur *et al.* [31] explored whether radiation dose was lower during cardiologist- or radiographer-controlled radiation exposure and determined whether the grade of the cardiologist influences radiation dose. They found that cardiologist-operated exposure and senior cardiologists result in lower radiation doses during CA. Kuon *et al.* [94] varied the image intensifier entrance dose level in CA and found that with the exception of cases with special requirements, lower dose levels guarantee adequate image quality with reduced patient dose. Philippe *et al.* [64] and Sandborg *et al.* [41] compared the radial arterial approach to the femoral approach and found that radial approach yielded significantly higher patient dose. Tsapaki *et al.* [44, 95] and Trianni *et al.* [47] evaluated the dose performance of flat-panel systems compared to conventional image intensifier systems. They concluded that flat panel systems produce images of higher quality with lower entrance dose rates than image intensifier systems and thus dose reduction with flat panel systems is possible. However in clinical practise the final performance of flat panel systems in terms of patient dose could give opposite results. Davies *et al.* [73] studied patient dose levels before and after the introduction of a dose reduction regime in EF and RFA procedures and found a considerable overall reduction in DAP. The results of these studies are tabulated in Table **5**.

An interesting issue regarding patient dosimetry is the contribution of fluoroscopy and image acquisition in total patient exposure. A number of studies report separate DAP values for fluoroscopy and fluorography during CA and PCI procedures [35, 62, 63, 96]. Mean DAP for fluoroscopy and image acquisition calculated from the values reported in these studies is shown in Fig. (**4**). In both procedures image acquisition contributes more in patient exposure although this is more profound in CA. Therefore minimization of fluorography would potentially lead to considerable patient dose reduction.

Another interesting issue is the comparison of patient radiation doses during non-invasive examinations with patient radiation doses during conventional coronary angiography. Multi-slice computed tomography (MSCT) coronary angiography is currently considered as a promising non-invasive alternative to conventional angiography due to recent advantages in spatial and especially temporal resolution of the technique [97, 98]. However, radiation dose is a major concern for MSCT coronary angiography, especially in cases of repeated examinations or in particular subgroups of patients (for example young female patients) [99]. Some investigators have compared radiation dose exposure during MSCT and conventional coronary angiography. The results are tabulated in Table **6**.

All investigators conclude that mean effective dose for MSCT coronary angiography is significantly higher than that of conventional angiography. The organs receiving the highest equivalent doses in MSCT coronary angiography are the female breasts, lungs, liver and oesophagus [78]. Thus as MSCT cardiac scanners are becoming increasingly available, operators must be aware of the radiation doses, the factors that affect it and the importance of dose reduction techniques.

### Risk to Patients

Invasive cardiology procedures provide great diagnostic and therapeutic benefit to patients but also subject them to considerable radiation exposure. On average, a coronary angiography corresponds to a radiation exposure to the patient of about 300 chest x-rays, while coronary stent implantation corresponds to 1000 chest x-rays and a radiofrequency ablation procedure up to 1500 chest x-rays [6, 101]. It is estimated that radiation induced cases of cancer per year in the UK is 280 cases per million of coronary angiographies whereas for CT scans, screening mammography and chest X-rays the cases of radiation induced cancer is 60, 8 and 1 cases per million examinations respectively [102]. In general, the justification of IC procedures is evident since they permit patients to avoid complicated invasive surgery which some might not tolerate due to factors of age or pathology [7]. Radiation is one of the many hazards to which a patient undergoing an IC procedure is exposed and it is generally accepted that the radiological risk, although high, will always be lower than other risks involved in the procedure [103, 104]. Nevertheless, radiation risk to patient must always be a matter of concern since in some complex procedures, patient skin doses can cross the threshold of deterministic effects whereas the increment of stochastic effects probability should also be taken into account, especially in young patients.

During a IC procedure the patient skin dose may occasionally exceed the threshold of 2Gy above which transient erythema and skin burns are observed [105]. In procedures where prolonged fluoroscopy time is used or when fluoroscopy is performed in a single projection the danger of large peak skin doses is increased. To avoid radiation skin injuries it is necessary to keep the exposure doses as low as can be reasonably achieved [106, 107], and it is recommended to monitor entrance surface doses. Unfortunately, real time maximum dose monitoring of the skin is difficult to assess in clinical practice since it is impossible to predict the site of maximum exposure before the intervention commences. In the absence of a direct measurement, conversion factors published in the literature may be used to establish peak skin dose from DAP values for CA and PCI [30, 46, 65]. Regarding CA, published conversion factors are 3,8 mGy/Gy·cm^2 ^[65], 3,9 mGy/Gy·cm^2^ [46] and 4.3 mGy/Gy·cm^2^ [108] whereas regarding PCI published conversion factors are 8.1 mGy/Gy·cm^2^ [65], 8.7 mGy/Gy·cm^2 ^[108] and 9.7 mGy/Gy·cm^2 ^[46].

To assess the potential risk of stochastic effects such as cancer and leukaemia, the effective dose to the patient must be calculated [109]. Unfortunately, determining the effective dose in clinical practise is not straightforward, mainly because of the complexity of the x-ray beam geometry and field size, variations during the catheterization procedure, and the individual patient anatomy. However an excellent correlation between effective dose and DAP has been found based on phantom measurements and Monte Carlo simulations, indicating that using a simple conversion factor to estimate effective dose from DAP values is acceptable [49]. Published conversion factors are 0.183 mSv/ Gy·cm^2 ^[22], 0.185 mSv/ Gy·cm^2 ^[50], and 0.221 mSv/ Gy·cm^2 ^[18].

### Patient Dose Reduction

According to the ‘as low as reasonably achievable’ (ALARA) and optimization principles [2] it is necessary to minimize patient dose in order to outweigh the radiation risk by the benefit of the interventional procedure. Therefore efforts should be made to properly manage radiation exposure to the patient. The most evident approach in order to reduce patient dose is by minimizing the beam-on time both for fluoroscopy and acquisition [79]. This can be achieved through the practice of intermittent fluoroscopy (short bursts of beam-on time) rather than continuous fluoroscopy [110]. Radiation field should be minimized to include only the anatomic region of interest since proper collimation of the x-ray beam substantially decreases patient dose. The image intensifier should be positioned as close to the patient’s body as possible while the height of the table should be adjusted to keep the body of the patient as further away from the x-ray tube as possible [69]. Fluoroscopic systems providing pulsed-fluoroscopy mode are preferable since, compared to a non pulsed system, a system that pulses the beam at 12.5 frames/s can result to 80% less exposure [110]. The use of high-level control to improve image quality under specific circumstances by increasing the dose rate should be avoided as much as possible. The same applies for the use of magnification which also increases the dose received by the patient. On the contrary, last image hold, a feature which presents the last acquired fluoroscopic frame on the video monitor [79, 111], obviating the need for continuous fluoroscopy, reduces fluoroscopic time and should be used as much as possible. At cine mode, the lower setting of frames per second should be used whenever possible (e.g. 15 frames/s instead of 30 frames/s). During procedures that require long fluoroscopy time, if clinically feasible, changing the radiographic projection minimizes patient skin dose [79]. Inspection and quality control of both the dose levels and the image quality of fluoroscopic equipment should be routinely contacted in order to assure optimum performance [69, 79]. Finally motivation and training of all laboratory personnel including radiographers and nursing staff and the overall efficiency of the catheterization laboratory contribute to patient dose reduction.

## CONCLUSION

Patient radiation doses vary widely among the different interventional cardiology procedures but also among published studies. Discrepancies of the derived results are patient-, procedure-, physician-, and fluoroscopic equipment-related. Nevertheless, IC procedures can subject patients to considerable radiation doses and efforts to minimize patient exposure should always be undertaken.

## Figures and Tables

**Fig. (1):**
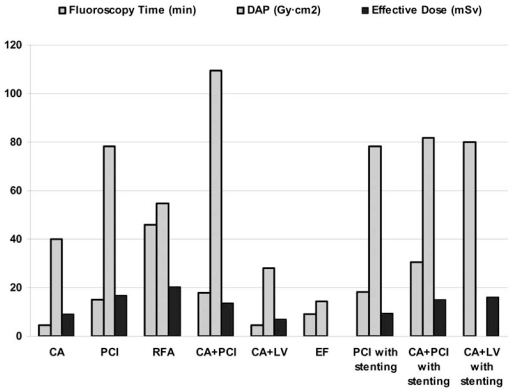
Mean fluoroscopy time, DAP and effective dose per type of intervention.

**Fig. (2):**
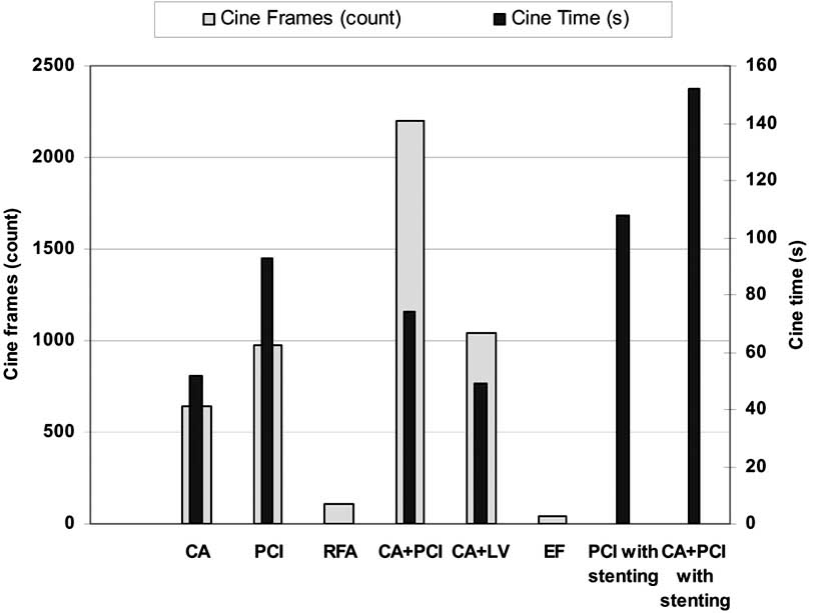
Mean cine frames and cine time per type of intervention.

**Fig. (3):**
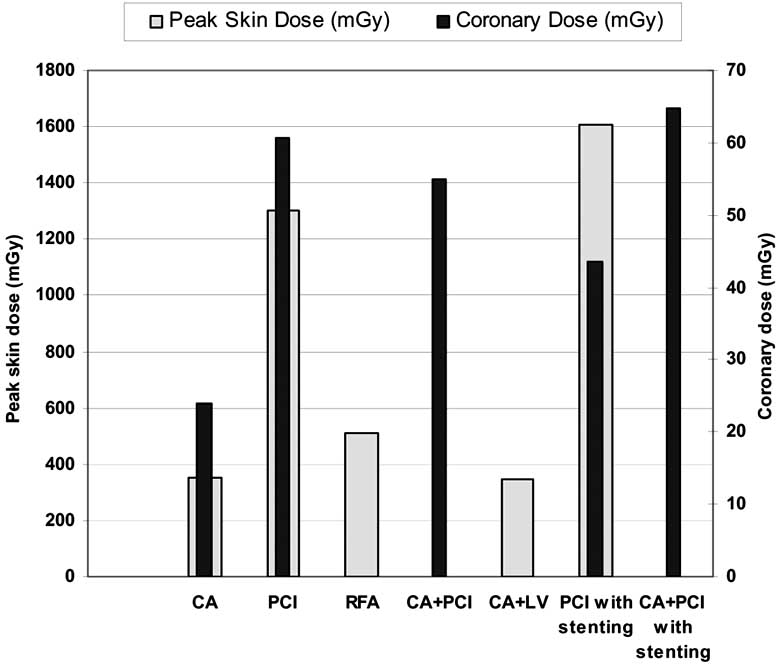
Mean peak skin dose and coronary dose per type of intervention.

**Fig. (4):**
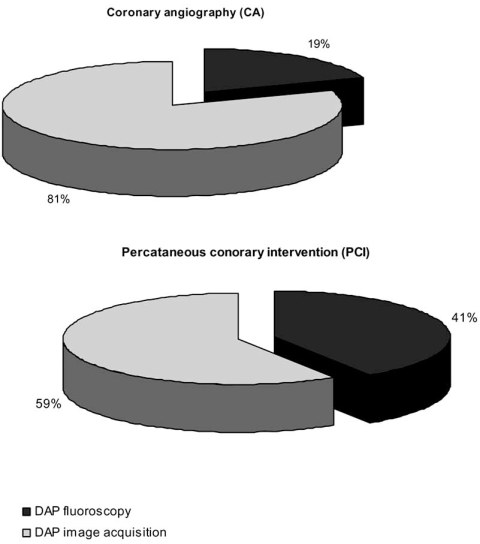
Contribution to total DAP of fluoroscopy and image acquisition during coronary angiography (CA) and percutaneous coronary intervention (PCI).

**Table 1:** Type of Procedures and Corresponding Number of Studies

Type of procedure	Number of studies	References
CA	47	[5, 8-53]
PCI	43	[5, 8-10, 12, 13, 15, 16, 19-22, 24, 25, 27, 28, 30, 32, 34, 35, 38, 40, 43-47, 49-51, 53-65]
RFA	17	[5, 20, 33, 45, 49, 53, 65-75]
CA+PCI	9	[14, 19, 22, 23, 25, 28, 41, 42, 49]
CA+LV	8	[24, 26, 33, 45, 54, 56, 58, 76]
EF	7	[33, 45, 49, 53, 71, 73, 74]
PCI with stenting	7	[22, 25, 27, 28, 33, 56, 58]
CA+PCI with stenting	2	[22, 28]
CA+LV+PCI with stenting	1	[76]

CA: coronary angiography; PCI: percutaneous coronary intervention; LV: left ventriculography; EF: electrophysiological study; RFA: radiofrequency ablation

**Table 2:** Assessed Quantities, Dosimetry Objective and Corresponding Number of Studies

Quantity	Dosimetry objective	No. of studies assessed this quantity
Fluoroscopy time*	Quality assurance	60
Dose Area Product (DAP)†	Quality assurance	53
Cine frames*	Quality assurance	27
Effective dose†	Stochastic risk	23
Cine time*	Quality assurance	12
Skin dose†	Deterministic risk	12
Coronary dose‡†	Deterministic risk	2

* non-dosimetric

† dosimetric

‡ dose to a coronary artery (in Gy) measured with a catheter based dosimeter during irradiation

**Table 3:** Dosimetric Measurements Per Type of Intervention

Examination	Total Sample	Fluoroscopy Time (min)			Cine Time (s)			Cine Frames (count)			DAP (Gy·cm^2^)			Peak Skin Dose (mGy)			Effective Dose (mSv)			Coronary Dose (mGy)		
		Average (*sample*)	Median (*sample*)	range	Average (sample)	Median (*sample*)	range	Average (sample)	Median (*sample*)	range	Average (sample)	Median (*sample*)	range	Average (sample)	Median (*sample*)	range	Average (sample)	Median (*sample*)	range	Average (sample)	Median(*sample*)	range
**CA**	9100	4.7 (*6398*)	4.1 (*1504*)	0.3-57	52 (*445*)	49 (*741*)	-	644 (*4122*)	1041 (*646*)	10-4490	39.9 (*7592*)	41.7 (*3365*)	1.1-2400	351 (*108*)	-	49-711	9.1 (*3418*)	-	0.3-15.8	24 (16)	-	-
**PCI**	5294	15 (*4273*)	11.1 (*645*)	1.4-172	93 (*1034*)	77 (*106*)	-	972 (*1749*)	1361 (*539*)	206-7969	78.3 (*4042*)	57.0 (*661*)	3.0-403.6	1304 (*253*)	-	170-1660	17.0 (*500*)	-	-	60.6 (291)	-	-
**RFA**	1682	45.8 (*1528*)	70.4 (*119*)	0.8-164	-	-	-	109 (*123*)	-	0-1270	54.6 (*407*)	46 (*285*)	0.03-430	513 (*132*)	-	3-3200	20.3 (*1043*)	-	-	-	-	-
**CA+PCI**	661	18 (*269*)	-	2.5-86	74 (125)	-	-	2199 (*78*)	-	360-6833	109.3 (*225*)	-	15.5-557.4	-	-	-	13.6 (*147*)	-	0.9-44.7	55 (7)	-	-
**CA+LV**	1297	4.6 (*1182*)	-	0.3-39	49 (*10*)	-	-	1038 (*106*)	-	470-1331	28.0 (*1176*)	20.0 (1139)	1.0-202.8	343.5 (*50*)	-	-	7^1^ (*100*)	-	-	-	-	-
**EF**	237	9.0 (*136*)	-	0.1-258	-	-	-	38 (*24*)	-	0-670	14.5 (*112*)	-	0.5-210	-	-	-	^-^	3.2 (*19*)	1.3-23.9	-	-	-
**PCI with stenting**	343	18.3 (*73*)	-	3-46	108 (*58*)	-	-	-	-	-	78.2 (*83*)	-	2.4-357	1606 (*35*)	-	-	9.6 (*29*)	-	-	43.6 (*15*)	-	-
**CA+PCI with stenting^2^**	16	30.4 (*16*)	-	-	152 (*16*)	-	-	-	-	-	81.8 (*16*)	-	-	-	-	-	15.1 (*16*)	-	-	64.7 (*9*)	-	-
**CA+LV+PCI with stenting^1^**	100	80 (*100*)	70 (*100*)	15-361	-	-	-	-	-	-	-	-	-	-	-	-	16 (*100*)	-	-	-	-	-

CA: coronary angiography, PCI: percutaneous transluminal coronary angioplasty, LV: left ventriculography, EF: electrophysiological study, RFA: radiofrequency ablation

1 One study reported results;

2 Two studies reported results

**Table 4:** Comparison of Results Between Studies Published Before and After the Year 2000

	Fluoroscopy Time (min)		DAP (Gy·cm^2^)		Effectime Dose (mSv)	
	*CA*	*PCI*	*CA*	*PCI*	*CA*	*PCI*
**before year 2000**	6.2	21.3	52.5	81.7	11.7	20
**after year 2000**	3.7	12.2	31.1	59.2	8.4	13.6

**Table 5:** DAP Values (cGy.cm^2^) Reported by Various Investigators. Evaluation of the Effect of Various Parameters on Patient Dose

		CA	PCI				CA	PCI
Broadhead *et al.* [20]	biplane system	47.7	72.2		Philippe *et al.* [64]	femoral approach		18.8
	single plane system	23.4	51.6			radial approach		28.6

Bernardi *et al.* [57]	simple procedure		65.8		Sandborg *et al.* [41]	femoral approach	38	47
	medium procedure		93			radial approach	51	75
	complex procedure		116.7					
					Tsapaki *et al.* [43]	image intensifier	39.3	44.3
Padovani *et al*. [60]	simple procedure		66.7			flat panel	51.1	
	medium procedure		96.4					
	complex procedure		132.7		Tsapaki *et al.* [44]	image intensifier	30	45
						flat panel	31	48
Arthur *et al.* [31]	cardiologist controlled radiation exposure	15.6						
	radiographer controlled radiation exposure	17.3			Traini *et al.* [47]	image intensifier	31.1	52
	cardiologist grade: first operator	13.6				flat panel	33.4	66.9
	cardiologist grade: assistant	20.8						

Kuon *et al.* [94]	dose level A (lowest)	5.97					**EF**	**RFA**
	dose levelB	6.73			Davies *et al.* [73]	standard dose	27.17	74.77
	dose level C	8.11				low dose	3.55	11.62
	dose level D (highest)	8.9						

**Table 6:** Comparison of Effective Dose Reported by Investigator Comparing Multislice Computed tomography Coronary Angiography (MSCT CA) with Conventional Coronary Angiography (CCA)

Study	Scanner	Effective dose (mSv)			
		MSCT CA	CCA	MSCT CA	CCA
		*patients without bypass grafts*		*patients with bypass grafts*	
Dill *et al.* [100]	16-slice	9.76±1.84	2.60±1.27	12.95±1.75	6.27±4.04
Coles *et al.* [48]	16-slice	14.7±2.2	5.6±2.7		
Jabara *et al.* [98]	64-slice			14.1±3.8	9.0±2.7

## References

[1] (2000). UNSCEAR. SOURCES AND EFFECTS OF IONIZING RADIATION, United Nations Scientific Committee on the Effects of Atomic Radiation UNSCEAR 2000 Report to the General Assembly, with scientific annexes.

[2] (1997). EURATOM. COUNCIL DIRECTIVE 97/43, On health protection of individuals against the dangers of ionising radiation in relation to medical exposure, and repealing Directive 84/466/Euratom. Official Journal of the European Communities.

[3] (1993). UNSCEAR, "Sources and effects of ionsing radiation. Report to the General Assembly, with scientific annexes.". New York: UN.

[4] (1990). NRPB. National Radiological Protection Board (NRPB) and Royal College of Radiologists (RCR). Patient dose reduction in diagnostic radiology. Report of the RCR and NRPB, Documents of the NRPB.

[5] Trianni A, Chizzola G, Toh H (2005). Patient skin dosimetry in haemodynamic and electrophysiology interventional cardiology. Radiat Prot Dosimetry.

[6] Picano E, Santoro G, Vano E (2007). Sustainability in the cardiac cath lab. Int J Cardiovasc Imaging.

[7] Baim D, Grossman W (1994). Cardiac Catheterization, Angiography, and Intervention Baltimore.

[8] Faulkner K, Love HG, Sweeney JK, Bardsley RA (1986). Radiation doses and somatic risk to patients during cardiac radiological procedures. Br J Radiol.

[9] Cascade PN, Peterson LE, Wajszczuk WJ, Mantel J (1987). Radiation exposure to patients undergoing percutaneous transluminal coronary angioplasty. Am J Cardiol.

[10] Holmes DR Jr, Wondrow MA, Gray JE (1990). Effect of pulsed progressive fluoroscopy on reduction of radiation dose in the cardiac catheterization laboratory. J Am Coll Cardiol.

[11] Coulden RA, Readman LP (1993). Coronary angiography: an analysis of radiographic practice in the UK. Br J Radiol.

[12] Pattee PL, Johns PC, Chambers RJ (1993). Radiation risk to patients from percutaneous transluminal coronary angioplasty. J Am Coll Cardiol.

[13] Dash H, Leaman DM (1984). Operator radiation exposure during percutaneous transluminal coronary angioplasty. J Am Coll Cardiol.

[14] Karpinnen J, Parviainen T, Servomaa A, Komppa K (1995). Radiation risk and exposure of radiologists and patients during coronary angiography and percutaneous transluminal angioplasty. Radiat Prot Dosimetry.

[15] Maccia C, Neofotistou V, Padovani R, Vano EWucherer M, Faulkner K, Teunen D (1995). "Patient doses in interventional radiology," in Radiation protection in interventional radiology.

[16] Vano E, Gonzalez L, Fernandez JM, Guibelalde E (1995). Patient dose values in interventional radiology. Br J Radiol.

[17] Kuon E, Lang E (1996). [Radiation burden in invasive cardiac diagnosis extent factors of influence and references for minimizing radiation dosage]. Z Kardiol.

[18] Leung KC, Martin CJ (1996). Effective doses for coronary angiography. Br J Radiol.

[19] Bakalyar DM, Castellani MD, Safian RD (1997). Radiation exposure to patients undergoing diagnostic and interventional cardiac catheterization procedures. Cathet Cardiovasc Diagn.

[20] Broadhead DA, Chapple CL, Faulkner K, Davies ML, McCallum H (1997). The impact of cardiology on the collective effective dose in the North of England. Br J Radiol.

[21] Zorzetto M, Bernardi G, Morocutti G, Fontanelli A (1997). Radiation exposure to patients and operators during diagnostic catheterization and coronary angioplasty. Cathet Cardiovasc Diagn.

[22] Betsou S, Efstathopoulos EP, Katritsis D, Faulkner K, Panayiotakis G (1998). Patient radiation doses during cardiac catheterization procedures. Br J Radiol.

[23] Neofotistou V (1997). Doses to patients and personnel during PTCA. Joint WHO/ISH Workshop on efficacy and radiation safety in interventional radiology.

[24] Padovani R, Novario R, Bernardi G (1998). Optimization in coronary angiography and percutaneous transluminar coronary angioplasty. Radiat Prot Dosimetry.

[25] Cusma JT, Bell MR, Wondrow MA, Taubel JP, Holmes DR Jr (1999). Real-time measurement of radiation exposure to patients during diagnostic coronary angiography and percutaneous interventional procedures. J Am Coll Cardiol.

[26] Clark AL, Brennan AG, Robertson LJ, McArthur JD (2000). Factors affecting patient radiation exposure during routine coronary angiography in a tertiary referral centre. Br J Radiol.

[27] Fransson SG, Persliden J (2000). Patient radiation exposure during coronary angiography and intervention. Acta Radiol.

[28] Katritsis D, Efstathopoulos E, Betsou S (2000). Radiation exposure of patients and coronary arteries in the stent era: A prospective study. Catheter Cardiovasc Interv.

[29] Lobotessi H, Karoussou A, Neofotistou V, Louisi A, Tsapaki V (2001). Effective dose to a patient undergoing coronary angiography. Radiat Prot Dosimetry.

[30] Vano E, Gonzalez L, Ten JI (2001). Skin dose and dose-area product values for interventional cardiology procedures. Br J Radiol.

[31] Arthur WR, Dhawan J, Norell MS, Hunter AJ, Clark AL (2002). Does cardiologist- or radiographer-operated fluoroscopy and image acquisition influence optimization of patient radiation exposure during routine coronary angiography?. Br J Radiol.

[32] Kuon E, Schmitt M, Dahm JB (2002). Significant reduction of radiation exposure to operator and staff during cardiac interventions by analysis of radiation leakage and improved lead shielding. Am J Cardiol.

[33] McFadden SL, Mooney RB, Shepherd PH (2002). X-ray dose and associated risks from radiofrequency catheter ablation procedures. Br J Radiol.

[34] Delichas MG, Psarrakos K, Molyvda-Athanassopoulou E (2003). Radiation doses to patients undergoing coronary angiography and percutaneous transluminal coronary angioplasty. Radiat Prot Dosimetry.

[35] Efstathopoulos EP, Makrygiannis SS, Kottou S (2003). Medical personnel and patient dosimetry during coronary angiography and intervention. Phys Med Biol.

[36] Hunold P, Vogt FM, Schmermund A (2003). Radiation exposure during cardiac CT: effective doses at multi-detector row CT and electron-beam CT. Radiology.

[37] Kuon E, Birkel J, Schmitt M, Dahm JB (2003). Radiation exposure benefit of a lead cap in invasive cardiology. Heart.

[38] Kuon E, Glaser C, Dahm JB (2003). Effective techniques for reduction of radiation dosage to patients undergoing invasive cardiac procedures. Br J Radiol.

[39] Kuon E, Gunther M, Gefeller O, Dahm JB (2003). Standardization of occupational dose to patient DAP enables reliable assessment of radiation-protection devices in invasive cardiology. Rofo.

[40] Tsapaki V, Kottou S, Vano E (2003). Patient dose values in a dedicated Greek cardiac centre. Br J Radiol.

[41] Sandborg M, Fransson SG, Pettersson H (2004). Evaluation of patient-absorbed doses during coronary angiography and intervention by femoral and radial artery access. Eur Radiol.

[42] Stisova V (2004). Effective dose to patient during cardiac interventional procedures (Prague workplaces). Radiat Prot Dosimetry.

[43] Tsapaki V, Kottou S, Kollaros N (2004). Comparison of a conventional and a flat-panel digital system in interventional cardiology procedures. Br J Radiol.

[44] Tsapaki V, Kottou S, Kollaros N (2004). Dose performance evaluation of a charge coupled device and a flat-panel digital fluoroscopy system recently installed in an interventional cardiology laboratory. Radiat Prot Dosimetry.

[45] Dragusin O, Desmet W, Heidbuchel H, Padovani R, Bosmans H (2005). Radiation dose levels during interventional cardiology procedures in a tertiary care hospital. Radiat Prot Dosimetry.

[46] Karambatsakidou A, Tornvall P, Saleh N (2005). Skin dose alarm levels in cardiac angiography procedures: is a single DAP value sufficient?. Br J Radiol.

[47] Trianni A, Bernardi G, Padovani R (2005). Are new technologies always reducing patient doses in cardiac procedures?. Radiat Prot Dosimetry.

[48] Coles DR, Smail MA, Negus IS (2006). Comparison of radiation doses from multislice computed tomography coronary angiography and conventional diagnostic angiography. J Am Coll Cardiol.

[49] Aroua A, Rickli H, Stauffer JC (2007). How to set up and apply reference levels in fluoroscopy at a national level. Eur Radiol.

[50] Bogaert E, Bacher K, Thierens H (2007). A Large-Scale Multicentre Study in Belgium of Dose Area Product Values and Effective Doses in Interventional Cardiology Using Contemporary X-Ray Equipment. Radiat Prot Dosimetry.

[51] Faj D, Steiner R, Trifunovic D (2007). Patient Dosimetry in Interventional Cardiology at the University Hospital of Osijek. Radiat Prot Dosimetry.

[52] Vijayalakshmi K, Kelly D, Chapple CL (2007). Cardiac catheterisation: radiation doses and lifetime risk of malignancy. Heart.

[53] Tsapaki V, Patsilinakos S, Voudris V (2008). Level of patient and operator dose in the largest cardiac centre in Greece. Radiat Prot Dosimetry.

[54] Finci L, Meier B, Steffenino G, Roy P, Rutishauser W (1987). Radiation exposure during diagnostic catheterization and single- and double-vessel percutaneous transluminal coronary angioplasty. Am J Cardiol.

[55] Federman J, Bell MR, Wondrow MA, Grill DE, Holmes DR Jr (1994). Does the use of new intracoronary interventional devices prolong radiation exposure in the cardiac catheterization laboratory?. J Am Coll Cardiol.

[56] Hwang E, Gaxiola E, Vlietstra RE (1998). Real-time measurement of skin radiation during cardiac catheterization. Cathet Cardiovasc Diagn.

[57] Bernardi G, Padovani R, Morocutti G (2000). Clinical and technical determinants of the complexity of percutaneous transluminal coronary angioplasty procedures: analysis in relation to radiation exposure parameters. Catheter Cardiovasc Interv.

[58] van de Putte S, Verhaegen F, Taeymans Y, Thierens H (2000). Correlation of patient skin doses in cardiac interventional radiology with dose-area product. Br J Radiol.

[59] Koenig TR, Wolff D, Mettler FA, Wagner LK (2001). Skin injuries from fluoroscopically guided procedures: part 1, characteristics of radiation injury. AJR Am J Roentgenol.

[60] Padovani R, Bernardi G, Malisan MR (2001). Patient dose related to the complexity of interventional cardiology procedures. Radiat Prot Dosimetry.

[61] Kuon E, Dahm JB, Schmitt M (2003). Short communication: time of day influences patient radiation exposure from percutaneous cardiac interventions. Br J Radiol.

[62] Efstathopoulos EP, Karvouni E, Kottou S (2004). Patient dosimetry during coronary interventions: a comprehensive analysis. Am Heart J.

[63] Kuon E, Empen K, Rohde D, Dahm JB (2004). Radiation exposure to patients undergoing percutaneous coronary interventions: are current reference values too high?. Herz.

[64] Philippe F, Larrazet F, Meziane T, Dibie A (2004). Comparison of transradial vs. transfemoral approach in the treatment of acute myocardial infarction with primary angioplasty and abciximab. Catheter Cardiovasc Interv.

[65] Chida K, Saito H, Otani H (2006). Relationship between fluoroscopic time, dose-area product, body weight, and maximum radiation skin dose in cardiac interventional procedures. AJR Am J Roentgenol.

[66] Calkins H, Niklason L, Sousa J (1991). Radiation exposure during radiofrequency catheter ablation of accessory atrioventricular connections. Circulation.

[67] Lindsay BD, Eichling JO, Ambos HD, Cain ME (1992). Radiation exposure to patients and medical personnel during radiofrequency catheter ablation for supraventricular tachycardia. Am J Cardiol.

[68] Kovoor P, Ricciardello M, Collins L, Uther JB, Ross DL (1998). Risk to patients from radiation associated with radiofrequency ablation for supraventricular tachycardia. Circulation.

[69] Perisinakis K, Damilakis J, Theocharopoulos N (2001). Accurate assessment of patient effective radiation dose and associated detriment risk from radiofrequency catheter ablation procedures. Circulation.

[70] Rosenthal LS, Mahesh M, Beck TJ (1998). Predictors of fluoroscopy time and estimated radiation exposure during radiofrequency catheter ablation procedures. Am J Cardiol.

[71] Paisey JR, Yue AM, White A (2004). Radiation peak skin dose to risk stratify electrophysiological procedures for deterministic skin damage. Int J Cardiovasc Imaging.

[72] Delle Canne S, Carosi A, Bufacchi A (2006). Use of GAFCHROMIC XR type R films for skin-dose measurements in interventional radiology. Validation of a dosimetric procedure on a sample of patients undergone interventional cardiology. Phys Med.

[73] Davies AG, Cowen AR, Kengyelics SM (2006). X-ray dose reduction in fluoroscopically guided electrophysiology procedures. Pacing Clin Electrophysiol.

[74] Efstathopoulos EP, Katritsis DG, Kottou S (2006). Patient and staff radiation dosimetry during cardiac electrophysiology studies and catheter ablation procedures: a comprehensive analysis. Europace.

[75] Kerzner R, Sanchez JM, Osborn JL (2006). Radiofrequency ablation of atrioventricular nodal reentrant tachycardia using a novel magnetic guidance system compared with a conventional approach. Heart Rhythm.

[76] Kocinaj D, Cioppa A, Ambrosini G (2006). Radiation dose exposure during cardiac and peripheral arteries catheterisation. Int J Cardiol.

[77] Balter S (2006). Methods for measuring fluoroscopic skin dose. Pediatr Radiol.

[78] Einstein AJ, Moser KW, Thompson RC, Cerqueira MD, Henzlova MJ (2007). Radiation dose to patients from cardiac diagnostic imaging. Circulation.

[79] Hirshfeld JW Jr, Balter S, Brinker JA (2005). ACCF/AHA/HRS/SCAI clinical competence statement on physician knowledge to optimize patient safety and image quality in fluoroscopically guided invasive cardiovascular procedures: a report of the American College of Cardiology Foundation/ American Heart Association/American College of Physicians Task Force on Clinical Competence and Training. Circulation.

[80] (2007). ICPR. Recomendations of the International Commision or Radiological Protection. Ann ICRP ICRP Publ 103.

[81] Hart D, Jones DL, Wall B (1994). "Estimation of effective dose in diagnostic radiology from entrance surface dose-area product measurements,".

[82] Rannikko S, Ermakov I, Lampinen JS (1997). Computing patient doses of X-ray examinations using a patient size- and sex-adjustable phantom. Br J Radiol.

[83] Gosch D, Gosch K, Kahn T (2007). [Conversion coefficients for estimation of effective dose to patients from dose area product during fluoroscopy x-ray examinations]. Rofo.

[84] Hart D, Jones DG, Wall BF (1994). "Estimation of effective doses in diagnostic radiology from entrance surface dose and dose-area product measurements,".

[85] Padovani R, Quai E (2005). Patient dosimetry approaches in interventional cardiology and literature dose data review. Radiat Prot Dosimetry.

[86] Chida K, Fuda K, Saito H (2007). Patient skin dose in cardiac interventional procedures: conventional fluoroscopy versus pulsed fluoroscopy. Catheter Cardiovasc Interv.

[87] Koval TM (1995). Potential iatrogenic component to coronary artery restenosis. Circulation.

[88] Balter S, Miller DL, Vano E (2008). A pilot study exploring the possibility of establishing guidance levels in x-ray directed interventional procedures. Med Phys.

[89] Bell MR, Berger PB, Menke KK, Holmes DR Jr (1992). Balloon angioplasty of chronic total coronary artery occlusions: what does it cost in radiation exposure, time, and materials?. Cathet Cardiovasc Diagn.

[90] Faulkner K, Marshall N, Lecomber A, Kotre C (1998). Establishment of Reference Doses for Examiniations Using Digital Fluoroscopy. Radiat Prot Dosimetry.

[91] Schwartz RS, Koval TM, Edwards WD (1992). Effect of external beam irradiation on neointimal hyperplasia after experimental coronary artery injury. J Am Coll Cardiol.

[92] Hehrlein. C/ Kaiser S, Riessen R (1999). External beam radiation after stent implantation increases neointimal hyperplasia by augmenting smooth muscle cell proliferation and extracellular matrix accumulation. J Am Coll Cardiol.

[93] Efstathopoulos E, Karvouni E, Kottou S (2004). Patient dosimetry during coronary interventions: a comprehensive analysis. Am Heart J.

[94] Kuon E, Dorn C, Schmitt M, Dahm JB (2003). Radiation dose reduction in invasive cardiology by restriction to adequate instead of optimized picture quality. Health Phys.

[95] Tsapaki V, Kottou S, Kollaros N, Kyriakidis Z, Neofotistou V (2005). Comparison of a CCD and a flat-panel digital system in an Interventional Cardiology Laboratory. Radiat Prot Dosimetry.

[96] Harrison D, Ricciardello M, Collins L (1998). Evaluation of radiation dose and risk to the patient from coronary angiography. Aust N Z J Med.

[97] Achenbach S, Daniel WG (2007). Current role of cardiac computed tomography. Herz.

[98] Jabara R, Chronos N, Klein L (2007). Comparison of multidetector 64-slice computed tomographic angiography to coronary angiography to assess the patency of coronary artery bypass grafts. Am J Cardiol.

[99] Paul JF, Abada HT (2007). Strategies for reduction of radiation dose in cardiac multislice CT. Eur Radiol.

[100] Dill T, Deetjen A, Ekinci O (2008). Radiation dose exposure in multislice computed tomography of the coronaries in comparison with conventional coronary angiography. Int J Cardiol.

[101] Picano E (2004). Informed consent and communication of risk from radiological and nuclear medicine examinations: how to escape from a communication inferno. BMJ.

[102] Berrington de Gonzalez A, Darby S (2004). Risk of cancer from diagnostic X-rays: estimates for the UK and 14 other countries. Lancet.

[103] Vano E, Arranz L, Sastre JM (1998). Dosimetric and radiation protection considerations based on some cases of patient skin injuries in interventional cardiology. Br J Radiol.

[104] Joint WHO ISH CE Workshop, "Efficacy and radiation safety in interventional radiology," Germany Bundesamt fur Starhlenchutz Munich-Germany BfS-ISH-178/97, 1197.

[105] Wagner LK, Eifel PJ, Geise RA (1994). Potential biological effects following high X-ray dose interventional procedures. J Vasc Interv Radiol.

[106] Willis CE, Slovis TL (2005). The ALARA concept in pediatric CR and DR: dose reduction in pediatric radiographic exams-a white paper conference. AJR Am J Roentgenol.

[107] Limacher MC, Douglas PS, Germano G (1998). ACC expert consensus document. Radiation safety in the practice of cardiology. American College of Cardiology. J Am Coll Cardiol.

[108] Quai E, Padovani R, Peterzol A (2003). Maximum skin dose assessment in interventional cardiology: results in three different European hospitals. Eur Radiol.

[109] (1990). ICRP. Recommendations of the International Commission on Radiological Protection. ICRP Publication 60. Ann ICRP.

[110] Weiss EM, Thabit O (2007). Clinical considerations for allied professionals: radiation safety and protection in the electrophysiology lab. Heart Rhythm.

[111] Seeram E, Travis E (1997). Radiation protection.

